# Web-Based Health Intervention for Young People Who Have a Parent with a Mental Illness: Delphi Study Among Potential Future Users

**DOI:** 10.2196/10158

**Published:** 2018-10-31

**Authors:** Jodie L Matar, Darryl J Maybery, Louise A McLean, Andrea Reupert

**Affiliations:** 1 Faculty of Education Monash University Clayton Australia; 2 Department of Rural Health Monash University Moe Australia; 3 Education, Psychology Programs Monash University Clayton Australia

**Keywords:** Delphi studies, early medical intervention, internet, preventative health, Web-based intervention, youth

## Abstract

**Background:**

Young people who have a parent with a mental illness face elevated risks to their mental health and well-being. However, they may not have access to appropriate interventions. Web-based interventions may reach and meet the needs of this at-risk group, yet their preferences regarding the features of this medium are unknown.

**Objective:**

This study sought to determine the utility of a Web-based intervention to meet the needs of young people who have a parent with a mental illness and their perspectives regarding the types of features of such a website.

**Methods:**

A systematic, 2-round Delphi study was employed to solicit the views of 282 young people aged 16 to 21 years (Round 1, n=14; Round 2, n=268) from urban and regional settings in Australia who self-reported that their parent has a mental illness. “Regional” was used to refer to nonurban participants in the study. After ascertaining whether a Web-based intervention was warranted, Web-based intervention features were identified, including how the site might be facilitated, topics, duration and frequency, and the nature of the professional contact. The extent to which young people agreed on the importance of these factors was assessed. Differences and similarities across gender and location were investigated. A mixed method analytic framework was employed using thematic analysis as well as 2-way between-groups analysis of covariance (ANCOVA) controlling for age and chi-square test of independence analysis.

**Results:**

Both rounds highlighted a strong preference for a Web-based intervention. Consensus was reached for a professionally monitored site, young people and professionals having equal input into the weekly facilitated sessions (eg, sharing the lead role in discussions or deciding on relevant session content), unlimited time access, 1-hour, open discussion, weekly sessions over 6 weeks, psychoeducation about mental illness, and considerations for the management of safety violations. There were significant main effects of location type and several of the preferred features for a Web-based intervention for young people who have a parent with a mental illness. However, effect sizes were small to moderate, limiting practical application.

**Conclusions:**

Young people aged 16 to 21 years indicated a need for a professionally monitored, psychoeducational, Web-based intervention, with input from professional facilitators and other young people who have a parent with a mental illness, in addition to recommendations to external resources. These findings may inform the development of future Web-based interventions for this highly vulnerable group.

## Introduction

The transition to adulthood can be a period marked by the onset of serious mental illness. While early intervention can reduce the severity and persistence of these illnesses, treatment is often delayed or not provided, resulting in a potentially avoidable disease burden [1]. Young people who have a parent with a mental illness are a particularly at-risk group, whose risk of developing mental illness ranges from 41% to 77% [2]. Web-based preventative and early interventions hold great promise. However, young people’s preferences on how such interventions might meet their needs have not been explored. This study aimed to identify whether these young people want a Web-based intervention and, if so, their perspectives on how it might be delivered and their preferred features.

Compared to their same-aged peers, young people who have a parent with a mental illness face an increased risk of acquiring a substance use disorder or mental illness, academic failure, and developing stress-related somatic health conditions such as asthma [3,4]. These young people may take on caring responsibilities for their parent or siblings [5]. In Australia, 21% to 23% of young people are estimated to have at least 1 parent with a mental illness [6]. Hence, these young people are a prevalent, high-risk group that warrant effective intervention to prevent or reduce the increased risks of adverse outcomes associated with parental mental illness.

Interventions for families impacted by parental mental illness have been shown to be effective. Siegenthaler, Munder, and Egger [7] found that interventions developed for this group decreased young people’s risk of developing mental illness by up to 40%. However, most interventions target the parent; for example, *Let’s Talk about Children* involves a clinician working with a parent to promote parenting competence and confidence within the context of their illness [8]. Yet, some parents do not seek help because they are in hospital, they do not acknowledge the impact of their illness on children, or are reluctant to disclose their parenting responsibilities because of the fear of losing their children to child protection authorities [8-10]. Some interventions exclude parents who are very ill or persistently use substances [8]. Consequently, the most vulnerable young people may miss out on essential services [11].

There are some youth-specific interventions for parental mental illness that aim to present psychoeducation, provide respite from caring responsibilities, and promote peer connectedness [12]. However, many peer support interventions lack a strong theoretical framework [13], and the evaluation undertaken is generally of relatively poor quality [14]. Many interventions for children in these families have age limits for participation (eg, only for those aged under 18) or exclude young people who have their own mental health issues. Additionally, reliance on public transport, lack of time, parental consent and, especially in regional areas, geographic constraints may impede young people’s attendance [15]. Given the stigma associated with mental illness [16], some young people in these families prefer anonymous services such as helplines [17].

To succeed in identifying and supporting young people who have a parent with a mental illness, services need to engage with young people in environments where they seek help and interact [18]. Young people are increasingly turning to the internet to find mental health information and support [19]. In Canada, Wetterlin et al [20] found that 61.6% of 521 young people aged between 17 to 24 years had utilized the internet to access information or seek help for how they were feeling, and 82.9% indicated that they were likely to use a mental health website to find information in challenging times. In a recent review on the use of Web-based and social networking interventions, it was recommended that services promote the use of Web-based technologies given the clear benefits in reducing symptomology (eg, depression) in young people [21]. The internet is appealing to young people because it is anonymous and may be easily accessed at all times of the day [22]. In particular, Web-based interventions may reach a more diverse population than traditional face-to-face interventions [23], including young people who might otherwise avoid services [16]. Grové et al [24] found a strong preference for Web-based support among young people whose parents have a mental illness. Thus, a Web-based medium may be a useful and age-appropriate platform in which to engage and support young people who have a parent with a mental illness.

There are some Web-based interventions specifically designed for this group of young people, including *Survivalkid* [22,25], *Grubbel* [26], and *Kopstoring* [27], although more research is needed on the evaluation and suitability of Web-based platforms for this at-risk target group. When developing mental health interventions, it is important to consult with end users [28]. While some studies have sought views from parents or professionals to ascertain what young people need [29], young people themselves often have differing opinions compared with adults [30]. Hence, it is crucial to solicit young peoples’ views on whether they want Web-based interventions and how such a medium may function. Capturing these preferences at the outset of intervention development will ensure that the resulting Web-based intervention is responsive to their needs and likely to be utilized [28].

The aim of this study was to explore the views of young people aged 16 to 21 years who have a parent with a mental illness regarding Web-based interventions. Specific research questions were: (1) Is a Web-based intervention a beneficial way to support this target group? If so, (2) How might a Web-based intervention be facilitated and monitored? (3) What features should be included in a Web-based intervention? (4) What topics (if any) should be included in a Web-based intervention? (5) What safety issues (if any) are there in providing a Web-based approach, and how this might be managed?

As females have been shown to be more interested in Web-based interventions and more likely to seek help compared with males [31], and location may make a difference to how young people interact with Web-based interventions [32], this study examined potential gender and location differences.

## Methods

### Research Design

The study employed a 2-round Delphi study with a panel of young people who have a parent with a mental illness. The Delphi approach is a structured method to derive consensus on issues for which evidence is scarce. It involves a panel of experts to elicit their direction on the topic. In this study, the panel of experts was young people with lived experience of having a parent with a mental illness [33]. The approach guaranteed participants’ anonymity, thereby preventing possible biases. This is important when dealing with sensitive topics such as mental illness [33].

### Participants

Young people residing in Australia, aged 16-21 years (inclusive), fluent in English, with a parent or parents with a mental illness (self-reported or diagnosed) were eligible to participate. Participants were from urban or regional settings. “Regional” was used to refer to nonurban participants in the study (ie, inner regional, outer regional, rural, remote, or very remote). Young people aged under 18 years required parental permission to participate. Participants were not required to participate in both rounds.

Recruitment for both rounds of the Web-based questionnaire was conducted via the researchers’ professional networks and through organizations designed for young people (eg, help lines or young people’s carers groups). The response rate is not reported, as the number of people who received the anonymous link is unknown. All Round 1 participants were invited to participate in Round 2. Participants were paid Aus $20 for their involvement in each round.

#### Delphi Round 1

In total, 33% (14/43) of the young people who commenced Round 1 were eligible. Reasons for exclusion from Round 1 were incomplete questionnaires (22/43, 51%), failure to meet age criteria (5/43, 12%), and currently living outside of Australia (3/43, 7%). Round 1 participants were aged 16-21 years, mean 19.0 (SD 1.8) years, with no participants aged 17 years. Of them, 64% (9/14) participants were female and 64% (9/14) were from urban locations.

#### Delphi Round 2

In Round 2, 99.3% (268/270) of the participants, aged 16-21 years, mean 19.4 (SD 1.2) years, were eligible to be included. In total, 28% (12/43) of the participants from Round 1 (ie, who partially or fully completed the questionnaire) participated in Round 2. Other participants for Round 2 (256/268, 95.5%) were invited via professional networks to participate in this study via an anonymous link. Of them, 0.8% (2/268) of the participants were excluded as one was too young and the other was living outside of Australia. Participants aged 20 years (99/268, 36.7%) represented the largest age group. While the largest number of participants were from urban locations (119/268, 44.4%), the representation was more widespread compared to Round 1.

Participants self-reported their parent’s main mental illness(es) aligned with the Diagnostic and Statistical Manual of Mental Disorders 5^th^ edition [34]. Parental mental illnesses in Round 1 and Round 2 represented 25% (5/20) and 55% (11/20) of the Diagnostic and Statistical Manual of Mental Disorders 5^th^ edition [34] classification chapters, respectively. Depressive and anxiety disorders were most commonly reported.

### Materials and Procedure

The study was approved by the Monash University Human Research Ethics committee. Web-based explanatory statements were provided to participants prior to each round. The questionnaire for Round 1 was based on the research questions and consisted of 22 questions in total, mainly of open-ended responses congruent with the Delphi method [35]. Questions related to different features of a Web-based intervention for young people who have a parent with a mental illness. Round 1 comprised of 14 open-ended questions related to intervention facilitation, interaction with peers and how this may function, preferences regarding communicating with a clinician, possible topics of interest, and safety issues and how such issues may be managed. The Round 1 questionnaire posed a closed question regarding whether a Web-based approach was a useful way to support young people who have a parent with a mental illness, along with an opportunity to provide reasons for a yes or no response.

The Round 2 questionnaire was developed from the themes identified in Round 1. It consisted of 39 questions related to the preferred structure of a Web-based intervention, peer interaction, whether the intervention should be time-limited, and preferred frequency and length of clinician-facilitated sessions. There were another 24 questions that required participants to rate the level of importance of the various features identified in Round 1 on an 11-point Likert-type scale from 0 (not important) to 10 (extremely important). These features related to the benefits of accessing a Web-based intervention, topics for Web-based intervention sessions, how Web-based interventions should be facilitated and monitored, internet safety, and issues around ethical issues (eg, informed consent). As discussed in preliminary data analyses, a consensus was reached after 2 rounds of the Delphi method.

### Preliminary Data Analyses

Data were collected using the Qualtrics Web-based questionnaire package and analyzed using IBM SPSS Statistics version 24. Delphi studies typically attract similar, smaller numbers of participants in each round (eg, 12-30 participants). However, the Round 2 participant numbers far exceeded those of Round 1, thus providing an opportunity to examine potentially important differences in participant subgroups according to age, gender, and location.

The Braun and Clarke [36] 6-step analytic approach was employed to analyze Round 1 Delphi data. Themes were identified independently by 2 researchers. The researchers then compared and contrasted their interpretations of the main themes by referring back to participants’ responses, to reach a consensus through discussion rather than a numerical level of agreement.

The Round 2 analysis included a rank ordering of preferences, frequencies, and the use of analysis of covariance (ANCOVA) and chi-square test of independence to identify differences across gender and urban and regional locations. In apriori correlations, age was found to be positively correlated with almost all variables, with older participants scoring more highly than those younger. This was subsequently controlled for in the ANCOVAs reported in the results section. Prior to analysis, data were examined to ensure that assumptions pertaining to each of the tests were met, with no violations encountered. Due to the exploratory nature of the analyses, Bonferroni corrections were not adopted (the likelihood of this increasing the type 1 errors is acknowledged) [37], and given the large amount of data generated in Round 2, only the key findings are reported here.

## Results

For ease of reporting, Round 1 and Round 2 findings are combined. Descriptive statistics for themes are shown in Table 1, with Round 1 themes in the left-hand column in Tables 2, 3, and 4. The tables are stratified by gender and location. Significant differences, significant interactions between gender and location on themes, and main effects are indicated in the tables and discussed in the text.

All participants in Round 1 (14/14, 100%) agreed that a Web-based intervention was a beneficial way to support young people who have a parent with a mental illness. This was extended in Round 2, where participants rated access to a Web-based intervention as very important (mean 8.52) on the 11-point (0-10) scale. The ANCOVA showed no main or interaction effects for gender or location.

In Round 1, 93% (13/14) participants agreed that a clinician should monitor a Web-based intervention group. In Round 2, participants rated it very important for a clinician to monitor a Web-based intervention (mean 8.01). The ANCOVA revealed neither interaction effect nor main effect for gender; however, there was a significant main effect for location, *F*_1, 240_=7.44, *P*=.01, partial η^2^=0.030. Urban participants rated a Web-based intervention facilitated by a clinician to be more important (mean 8.21) than regional participants (mean 7.88). Note, however, that both groups provided very high ratings.

Round 2 participants were asked for their preference for the duration (as a percentage) that they wanted a clinician and peers to lead discussions during each Web-based group session. No participant preferred 100% direction from either a clinician or peers. These preferences are shown in Table 1 and were analyzed further (ie, gender and location) using chi-square, with the only significant difference being that more urban young people reported that the approach should include weekly goals to practice between sessions compared with regional participants, χ^2^_1_=12.4, *P*<.001, phi=−0.266 (n=244).

As shown in Table 1, Round 2 participants preferred weekly sessions over 6 weeks, an “open and free discussion depending on the needs of the group that week” (ranked 1). Preferences about time limitations, length, frequency, and duration of individual sessions were analyzed further (ie, gender and location) using chi-square, with the only significant difference being that more urban young people reported that the approach should be unlimited in time, compared with regional participants, χ^2^_1_=5.2, *P*=.02, phi=0.155 (n=244).

Participants were then asked about the features that should be in a Web-based intervention. As shown in Table 2, Round 1 participants agreed there should be multiple features, and Round 2 participants rated these features as very important.

**Table 1:** Round 2 first preference rankings and participant frequency analysis for Web-based group intervention structure based on Round 1 themes.

Web-based intervention themes		Round 2 total (N=268)	Gender		Location type	
			Males (n=157)	Females (n=111)	Urban (n=119)	Regional (n=125)
**Web-based intervention structure, n (%)**						
	Unlimited timeframe preferred^a^	201 (75.0)	125 (79.6)	76 (68.5)	97 (81.5)	85 (68.0)
	6-week intervention preferred by participants who selected a time-limited intervention	27 (10.1)	14 (8.9)	13 (11.7)	8 (6.7)	19 (15.2)
	Weekly session frequency	171 (63.8)	98 (62.4)	73 (65.8)	77 (64.7)	80 (64.0)
	Duration of 1 hour	120 (44.8)	67 (42.7)	53 (47.7)	46 (38.7)	61 (48.8)
**Group session facilitation, n (%)**						
	Ratio of 50% by clinician and 50% by peer	138 (51.5)	76 (48.4)	62 (55.9)	67 (56.3)	56 (44.8)
**Group session structure (≥1 preferences allowed), n (%)**						
	Open and free discussion depending on needs of the group that week (ranking 1)	227 (84.7)	126 (80.3)	81 (73.0)	99 (83.2)	17 (13.6)
	Set topics each week (ranking 2)	207 (77.2)	134 (85.4)	93 (83.8)	93 (78.2)	92 (73.6)
	Weekly goals to work on and practice between sessions^a^ (ranking 3)	190 (70.9)	113 (72.0)	77 (69.4)	96 (80.7)	75 (60.0)

^a^Significant difference reported for location.

**Table 2:** Round 1 themes and Round 2 mean importance ratings and rankings of Round 1 themes for preferred features of a Web-based intervention.

Subthemes identified in Round 1		Round 2 importance rating (N=268)^a^	Location type (n=244)^b^	
			Urban (n=119)	Regional (n=125)
**Referral(s) to other sources for additional support^c^, mean (SD)**		7.84 (1.3)	N/A^d^	N/A
	Males	N/A	7.78 (1.4)	7.97 (1.0)
	Females	N/A	8.16 (1.3)	7.63 (1.2)
**Privately contact clinician from Web-based sessions^e^, mean (SD)**		7.81 (1.2)	N/A	N/A
	Males	N/A	7.72 (1.4)	7.73 (1.1)
	Females	N/A	8.08 (1.3)	7.73 (1.1)
**Open access to a chat room^c^, mean (SD)**		7.76 (1.3)	N/A	N/A
	Males	N/A	7.52 (1.6)	7.96 (0.9)
	Females	N/A	8.06 (1.4)	7.65 (1.3)
**Privately contact peers (Web-based intervention group), mean (SD)**		7.69 (1.4)	N/A	N/A
	Males	N/A	7.64 (1.5)	7.82 (1.2)
	Females	N/A	7.76 (1.5)	7.37 (1.4)
**Referral(s) to additional clinical support^e^, mean (SD)**		7.40 (1.2)	N/A	N/A
	Males	N/A	7.20 (1.3)	7.35 (1.0)
	Females	N/A	7.68 (1.4)	7.49 (1.2)
**Web-based chat room sessions with set topics decided by a clinician, mean (SD)**		7.31 (1.4)	N/A	N/A
	Males	N/A	7.22 (1.5)	7.57 (0.2)
	Females	N/A	7.16 (1.8)	7.31 (0.2)
**Young people and a clinician in a Web-based chat room session, mean (SD)**		7.24 (1.5)	N/A	N/A
	Males	N/A	6.99 (1.7)	7.41 (1.4)
	Females	N/A	7.54 (1.6)	7.29 (1.2)

^a^11-point Likert-type scale from 0 (not important) to 10 (extremely important).

^b^Males (143/244, 58.6%); females (101/244, 41.4%).

^c^Significant interaction reported between gender and location.

^d^N/A: not applicable.

^e^Significant interaction effects for gender.

The ANCOVA analysis identified a significant interaction between gender and location on referral(s) to other sources for additional support (*F*_1, 239_=5.05, *P*=.03, η^2^=0.021) and open access to a chat room (*F*_1, 239_=5.95, *P*=.02, η^2^=.024), with female urban participants reporting significantly higher scores for these preferred features (Table 2 for scores). Additionally, there were 2 significant interaction effects for gender for privately contacting a clinician from the Web-based sessions (*F*_1, 239_=4.66, *P*=.03, η^2^=0.019) and referral(s) to additional clinical support (*F*_1, 239_=7.18, *P*=.008, η^2^=0.029) with urban females scoring more highly for these preferred features (Table 2 for scores).

In Round 1, 79% (11/14) participants indicated that self-care, resilience building, ways of coping, psychoeducation, and how to ask for help and where to get help were important topics to include in a Web-based intervention. They also indicated a range of other topics that, for Round 2, were grouped into 4 common themes and 10 subthemes (Table 3). Table 3 shows the Round 1 topics and descriptive statistics, importance ratings, and rankings from Round 2.

While participants scored all themes and subthemes highly (at 7 and above), they scored highest in regard to helping and supporting a parent with a mental illness, useful ways to cope when parents have a mental illness, and psychoeducation around specific parental mental illness. A series of ANCOVA analyses were then undertaken with all but one (general mental illness) showing main effects for location, with urban participants having higher mean scores than their regional counterparts. For brevity, ANCOVA statistics are not shown here but are available from the authors. In regard to gender, there was only 1 variable (helping and supporting a parent with a mental illness) where males had higher mean scores than females (*F*-statistics and means not shown here).

**Table 3:** Round 1 topics of interest with Round 2 descriptive statistics, importance ratings, and rankings.

Themes and subthemes from Round 1 responses and Round 2 gender (males n=143, females n=101)			Importance (N=268)^a^		Location type (n=244)	
			Rating, mean (SD)	Rank	Urban (n=119), mean (SD)	Regional (n=125), mean (SD)
**Psychoeducation**						
	**Helping and supporting parent with a mental illness^b,c^**		8.69 (1.21)	1	N/A^d^	N/A
		Male	N/A	N/A	9.01 (1.0)	8.55 (0.9)
		Female	N/A	N/A	8.62 (1.6)	8.31 (1.3)
	**Specific parental mental illness^b^**		8.59 (1.14)	3	N/A	N/A
		Male	N/A	N/A	8.86 (1.0)	8.41 (1.0)
		Female	N/A	N/A	8.64 (1.4)	8.29 (2.0)
	**Personal mental health and related information^b^**		8.55 (1.20)	4	N/A	N/A
		Male	N/A	N/A	8.72 (1.2)	8.34 (1.1)
		Female	N/A	N/A	8.76 (1.4)	8.31 (1.1)
	**Dealing with situations arising from parental mental illness^b^**		7.92 (1.26)	8	N/A	N/A
		Male	N/A	N/A	8.20 (1.2)	7.69 (1.9)
		Female	N/A	N/A	8.26 (1.5)	7.61 (2.0)
	**General mental illness**		7.54 (1.86)	10	N/A	N/A
		Male	N/A	N/A	7.74 (1.9)	7.42 (1.7)
		Female	N/A	N/A	7.66 (2.1)	7.08 (1.8)
**Emotional well-being**						
	**Build resilience^b^**		8.54 (1.2)	5	N/A	N/A
		Male	N/A	N/A	8.72 (1.2)	8.41 (1.2)
		Female	N/A	N/A	8.70 (1.1)	8.14 (1.2)
	**Promote general emotional well-being^b^**		8.45 (1.4)	6	N/A	N/A
		Male	N/A	N/A	8.61 (1.6)	8.16 (1.3)
		Female	N/A	N/A	8.78 (1.2)	8.12 (1.3)
	**Self-care strategies^b^**		8.38 (1.3)	7	N/A	N/A
		Male	N/A	N/A	8.57 (1.3)	8.11 (1.1)
		Female	N/A	N/A	8.80 (1.2)	7.96 (1.5)
**Coping strategies and skills**						
	**Useful ways to cope when parents have a mental illness^b^**		8.65 (1.3)	2	N/A	N/A
		Male	N/A	N/A	8.90 (1.1)	8.43 (1.2)
		Female	N/A	N/A	8.82 (1.4)	8.31 (1.3)
**Other resources**						
	**Access to other resources besides the Web-based intervention^b^**		7.58 (1.1)	9	N/A	N/A
		Male	N/A	N/A	7.87 (1.1)	7.38 (1.0)
		Female	N/A	N/A	7.70 (1.5)	7.31 (.8)

^a^11-point Likert-type scale from 0 (not important) to 10 (extremely important).

^b^Main effect for location.

^c^Main effect for gender.

^d^N/A: not applicable.

**Table 4:** Round 1 themes and subthemes with Round 2 importance ratings and rankings for safety issues with a Web-based intervention.

Themes and subthemes from Round 1 responses and gender (males n=143, females n=101)			Round 2 importance (N=268)^a^				Location type (n=244)		
			Mean (SD)		Rank		Urban (n=119), mean (SD)		Regional (n=125), mean (SD)
**Knowledge of prohibition from Web-based** **intervention if safety guidelines are not adhered** **to**			7.60 (1.34)		1		N/A^b^		N/A
	Males	N/A		N/A		7.42 (1.53)		7.78 (1.13)	
	Females	N/A		N/A		7.70 (1.39)		7.69 (1.26)	
**Clinician monitoring for cyberbullying**			7.59 (1.47)		2		N/A		N/A
	Males	N/A		N/A		7.51 (1.61)		7.66 (1.19)	
	Females	N/A		N/A		7.84 (1.46)		7.63 (1.26)	
**Web-based communication**			7.53 (1.38)		3		N/A		N/A
	Males	N/A		N/A		7.41 (1.43)		7.55 (1.15)	
	Females	N/A		N/A		7.80 (1.47)		7.57 (1.53)	
**Participation without parental consent (n=56)**			6.48 (2.14)		N/A		N/A		N/A
	Males (n=26)	N/A		N/A		6.57 (1.3)		6.37 (1.2 )	
	Females (n=30)	N/A		N/A		6.80 (1.2)		6.70 (1.1)	

^a^11-point Likert-type scale from 0 (not important) to 10 (extremely important).

^b^N/A: not applicable.

Participants raised 2 potential safety and ethical issues, specifically in regard to informed consent. Participants did not want to have to obtain parental consent for several reasons: a perception that their parent would not understand why they sought help, a concern about embarrassing or disappointing their parent, fear of their parent’s reaction (eg, anger, guilt, or upset), worry that their parents would ban it, and a concern that by asking, they would strain the relationship with their parent, particularly if the parent with the mental illness was the only parent in the house. The key themes from Round 1 responses importance ratings and rankings for safety issues with a Web-based intervention are shown in Table 4. An ANCOVA revealed no interaction effect or main effects for gender or location.

## Discussion

### Principal Findings

The young people involved in this Delphi study (across age groups and locations) indicated a strong preference for a Web-based approach along with a range of preferred features. Developers should attempt to take these preferences into account when developing Web-based interventions for this target group. An especially important finding was young people’s preferences for a Web-based intervention that was facilitated by professionals. This preference has efficacy support; in a meta-analysis, Andersson and Cuijpers [38] found that Web-based interventions with professional support were much more effective than those without professional support in the treatment of adult depression.

Notwithstanding their preference for professional facilitation, young people preferred to have equal input during weekly sessions, with young people and professional facilitators sharing the role of directing discussions and contributing to session content. For example, a professional facilitator may allow young people to spontaneously direct discussions around their preferred content for up to half of the weekly session based on their needs or interest. Young people also indicated a preference for weekly, scheduled, 1-hour open discussion sessions, according to the particular needs of the group, as well as set topics. Hence, it appears to be important that Web-based interventions are sufficiently flexible to present established topics and be responsive to the immediate needs of young people participating in the intervention. The *Kopstoring* Web-based intervention has pre-established topics each week but does not appear to allow for open-ended discussion sessions [27]. Additionally, young people indicated a preference for weekly goals to work on between sessions. Others have also highlighted the importance of goal setting with these young people; one study found that through goal setting, young people who have a parent with a mental illness were more likely to achieve their personal goals, which included increasing their understanding of mental illness and improving family connections [39].

Psychoeducation was considered the most important topic to be covered by the intervention. In particular, young people identified a need to learn about their parent’s mental illness, how to support their parent, personal mental health, and how they might deal with situations arising from their parent’s illness. These findings respond to reports of young people’s limited understanding of their parent’s mental illness [40]. Psychoeducation is a key component of a range of interventions for families affected by parental mental illness [3,41] and resonates with previous findings regarding children’s requests to find out more about their parent’s illness [24,42]. Young people also wanted to learn adaptive coping strategies, which is important given the tendency for some young people in these families to employ coping strategies such as avoidance or isolation [43].

As well, young people indicated a preference for a time-unlimited intervention. If it had to be time-limited, young people preferred a 6-week Web-based intervention. Some Web-based interventions for this target group are offered weekly and are generally limited to 8 weeks [26,27]. Survivalkid, in the Netherlands, is unlimited but does not offer structured weekly topics [22]. Further research is required to ascertain the most effective length of Web-based interventions for this target group in terms of efficacy as well as participant satisfaction.

In Round 2, young people identified a need for referrals for additional support. Likewise, others have suggested that multiple interventions may need to be made available to this group of young people [44]. Empowering young people to access their own supports (rather than rely on their parents) potentially addresses the service gaps with identification, referrals, and fears around accessing help due to stigma [45]. Urban females scored higher than all others on referrals for support and wanting open access to chat rooms. This is not surprising given young female adults have reported themselves as their strongest help-seeking influence compared with other social influences, and this was higher for females compared with males [31]. Additionally, young people wanted the opportunity to privately chat with a Web-based facilitator. Survivalkid in the Netherlands has “survival coaches,” not therapists, who provide advice and may refer young people to more formal services if required [25]. Organizations would need to consider the viability of private contact given the time demands, staffing required, and costs involved.

Lastly, young people were asked to identify potential safety issues around providing a Web-based approach and invited to consider how this could then be managed. Young people wanted assurances that participants not adhering to guidelines would be banned or otherwise dealt with appropriately. Likewise, internet safety is an important consideration for young people when engaging in Web-based interventions [20,46]. Young people aged 16 and 17 indicated that they wanted the opportunity to participate in a Web-based intervention without having to obtain their parent’s consent, primarily because they were concerned about their parent’s reaction. Parents may act as “gatekeepers” for their children’s involvement in interventions [47], a concern aligned with the views of young people elicited here. Finally, it is a normal developmental phase for young adults to differentiate themselves from their parents [48], which again is well encapsulated in the preferences of young people in this study.

Generally, there were statistically significant main effects of location type between urban and regional young people. However, the practical significance [37] of this is limited given the small effect sizes and the observation that actual importance ratings for the different variables were still high for both urban and regional young people.

### Limitations

Several limitations apply in interpreting the findings. A smaller number of young people aged 16 and 17 years were recruited compared with the other age groups. Parental mental illnesses were self-reported by those surveyed rather than clinically diagnosed. It is unknown whether participants had their own mental health concerns, which may impact participants’ preferences for a Web-based intervention. The Web-based recruitment strategy might have favored those young people with an existing interest in and preference for Web-based supports. Future studies might differentiate between specific age groups to identify their respective needs for Web-based support, who might also be screened for their own mental health concerns. Consideration is also required to develop strategies for identifying young people at an earlier age. Working in collaboration with parents will be key here. Moreover, ongoing research is needed to investigate and evaluate the efficacy and effectiveness of Web-based interventions. The uptake of Web-based interventions also needs to be gauged, given that others have found that while young people prefer Web-based interventions, engagement is often low [20].

### Conclusions

This study constitutes a step in the development of Web-based interventions for young people who have a parent with a mental illness. Young people indicated a preference for a time-unlimited, Web-based intervention that was professionally monitored but with input from both young people and professionals. The results of this study might be used to inform the development of Web-based interventions in regard to topics, intervention length and moderation, and safety requirements. The value in seeking the views of those with lived experience is vital in the design of support. Web-based interventions hold promise to prevent or reduce the risk of adverse outcomes in young people into adulthood and potentially reduce the intergenerational risks of parental mental illness on future generations.

## Abbreviations

ANCOVA: analysis of covariance

## References

[1] McGorry PD, Hamilton MP (2016). Stepwise expansion of evidence-based care is needed for mental health reform. Med J Aust.

[2] Hosman CMH, van Doesum KTM, van Santvoort F (2014). Prevention of emotional problems and psychiatric risks in children of parents with a mental illness in the Netherlands: I. The scientific basis to a comprehensive approach. Australian e-Journal for the Advancement of Mental Health.

[3] Farahati F, Marcotte D, Wilcox-Gök V (2003). The effects of parents’ psychiatric disorders on children’s high school dropout. Economics of Education Review.

[4] Reupert A, Maybery D (2016). What do we know about families where parents have a mental illness? A systematic review. Child & Youth Services.

[5] Kallander EK, Weimand BM, Becker S, Van Roy B, Hanssen-Bauer K, Stavnes K, Faugli A, Kufås E, Ruud T (2017). Children with ill parents: extent and nature of caring activities. Scand J Caring Sci.

[6] Maybery DJ, Reupert AE, Patrick K, Goodyear M, Crase L (2018). Prevalence of parental mental illness in Australian families. Psychiatr. bull.

[7] Siegenthaler E, Munder T, Egger M (2012). Effect of preventive interventions in mentally ill parents on the mental health of the offspring: systematic review and meta-analysis. J Am Acad Child Adolesc Psychiatry.

[8] Solantaus T, Paavonen EJ, Toikka S, Punamäki R (2010). Preventive interventions in families with parental depression: children's psychosocial symptoms and prosocial behaviour. Eur Child Adolesc Psychiatry.

[9] Foster KP, Hills D, Foster KN (2018). Addressing the support needs of families during the acute hospitalization of a parent with mental illness: A narrative literature review. Int J Ment Health Nurs.

[10] David DH, Styron T, Davidson L (2011). Supported Parenting to Meet the Needs and Concerns of Mothers with Severe Mental Illness. Am J Psychiatr Rehabil.

[11] Potter R, Mars B, Eyre O, Legge S, Ford T, Sellers R, Craddock N, Rice F, Collishaw S, Thapar A, Thapar AK (2012). Missed opportunities: mental disorder in children of parents with depression. Br J Gen Pract.

[12] Foster K, McPhee I, Fethney J, McCloughen A (2014). Outcomes of the ON FIRE peer support programme for children and adolescents in families with mental health problems. Child &amp; Family Social Work.

[13] Reupert AE, Cuff R, Drost L, Foster K, van Doesum KTM, van Santvoort F (2013). Intervention programs for children whose parents have a mental illness: a review. Med J Aust.

[14] Bee P, Bower P, Byford S, Churchill R, Calam R, Stallard P, Pryjmachuk S, Berzins K, Cary M, Wan M, Abel K (2014). The clinical effectiveness, cost-effectiveness and acceptability of community-based interventions aimed at improving or maintaining quality of life in children of parents with serious mental illness: a systematic review. Health Technol Assess.

[15] Van Doesum KTM, Riebschleger J, Carroll J, Grové C, Lauritzen C, Mordoch E, Skerfving A (2016). Successful recruitment strategies for prevention programs targeting children of parents with mental health challenges: An international study. Child & Youth Services.

[16] Oh E, Jorm AF, Wright A (2009). Perceived helpfulness of websites for mental health information: a national survey of young Australians. Soc Psychiatry Psychiatr Epidemiol.

[17] Grové C, Reupert A, Maybery D (2015). Peer connections as an intervention with children of families where a parent has a mental illness: Moving towards an understanding of the processes of change. Children and Youth Services Review.

[18] Marcus MA, Westra HA, Eastwood JD, Barnes KL, Mobilizing Minds Research Group (2012). What are young adults saying about mental health? An analysis of Internet blogs. J Med Internet Res.

[19] Gowen LK (2013). Online Mental Health Information Seeking in Young Adults with Mental Health Challenges. Journal of Technology in Human Services.

[20] Wetterlin FM, Mar MY, Neilson EK, Werker GR, Krausz M (2014). eMental health experiences and expectations: a survey of youths' Web-based resource preferences in Canada. J Med Internet Res.

[21] Rice SM, Goodall J, Hetrick SE, Parker AG, Gilbertson T, Amminger GP, Davey CG, McGorry PD, Gleeson J, Alvarez-Jimenez M (2014). Online and social networking interventions for the treatment of depression in young people: a systematic review. J Med Internet Res.

[22] Drost LM, Cuijpers P, Schippers GM (2011). Developing an interactive website for adolescents with a mentally ill family member. Clin Child Psychol Psychiatry.

[23] Griffiths KM, Christensen H (2007). Internet-based mental health programs: a powerful tool in the rural medical kit. Aust J Rural Health.

[24] Grové C, Reupert A, Maybery D (2016). The Perspectives of Young People of Parents with a Mental Illness Regarding Preferred Interventions and Supports. J Child Fam Stud.

[25] Drost LM, Schippers GM (2015). Online support for children of parents suffering from mental illness: a case study. Clin Child Psychol Psychiatry.

[26] Elgán TH, Kartengren N, Strandberg AK, Ingemarson M, Hansson H, Zetterlind U, Gripenberg J (2016). A web-based group course intervention for 15-25-year-olds whose parents have substance use problems or mental illness: study protocol for a randomized controlled trial. BMC Public Health.

[27] Woolderink M, Smit F, van der Zanden R, Beecham J, Knapp M, Paulus A, Evers S (2010). Design of an internet-based health economic evaluation of a preventive group-intervention for children of parents with mental illness or substance use disorders. BMC Public Health.

[28] Trowse LC, Cook JG, Clooney TJA (2012). Mental health consumer and carer participation: why we bother. MJA Open.

[29] Jander A, Crutzen R, Mercken L, De Vries H (2015). Web-based interventions to decrease alcohol use in adolescents: a Delphi study about increasing effectiveness and reducing drop-out. BMC Public Health.

[30] Maybery D, Ling L, Szakacs E, Reupert A (2014). Children of a parent with a mental illness: perspectives on need. Australian e-Journal for the Advancement of Mental Health.

[31] Rickwood DJ, Mazzer KR, Telford NR (2015). Social influences on seeking help from mental health services, in-person and online, during adolescence and young adulthood. BMC Psychiatry.

[32] Griffiths KM, Mackinnon AJ, Crisp DA, Christensen H, Bennett K, Farrer L (2012). The effectiveness of an online support group for members of the community with depression: a randomised controlled trial. PLoS One.

[33] Hsu C, Sandford SB (2007). The Delphi technique: Making sense of consensus. Practical Assessment, Research & Evaluation.

[34] American Psychiatric Association (2013). Diagnostic and Statistical Manual of Mental Disorders.

[35] Custer RL, Scarcella JA, Stewart BR (1999). The modified Delphi technique: A rotational modification. Journal of Career and Technical Education.

[36] Braun V, Clarke V (2006). Using thematic analysis in psychology. Qualitative Research in Psychology.

[37] Gravetter FG, Wallnau LB (2009). Statistics for the Behavioural Sciences (8th ed). Statistics for the Behavioural Sciences.

[38] Andersson G, Cuijpers P (2009). Internet-based and other computerized psychological treatments for adult depression: a meta-analysis. Cogn Behav Ther.

[39] Maybery D, Reupert A, Goodyear M (2013). Goal setting in recovery: families where a parent has a mental illness or a dual diagnosis. Child & Family Social Work.

[40] Mordoch E (2010). How children understand parental mental illness: “you don't get life insurance. What's life insurance?”. J Can Acad Child Adolesc Psychiatry.

[41] Marston N, Stavnes K, Van Loon LMA, Drost LM, Maybery D, Mosek A, Nicholson J, Solantaus T, Reupert A (2016). A content analysis of Intervention Key Elements and Assessments (IKEA): What's in the black box in the interventions directed to families where a parent has a mental illness?. Child & Youth Services.

[42] Gladstone BM, Boydell KM, Seeman MV, McKeever PD (2011). Children's experiences of parental mental illness: a literature review. Early Interv Psychiatry.

[43] Holmila MJ, Itäpuisto M, Ilva M (2010). Invisible victims or competent agents: Opinions and ways of coping among children aged 12-18 years with problem drinking parents. Drugs: Education, Prevention and Policy.

[44] Steer S, Reupert A, Maybery D (2011). Programs for Children of Parents who have a Mental Illness: Referral and Assessment Practices. “One size fits all”?. Australian Social Work.

[45] Trondsen MV (2012). Living with a mentally ill parent: exploring adolescents' experiences and perspectives. Qual Health Res.

[46] Mar MY, Neilson EK, Torchalla I, Werker GR, Laing A, Krausz M (2014). Exploring e-Mental Health Preferences of Generation Y. Journal of Technology in Human Services.

[47] Reupert A, Goodyear M, Maybery D (2011). Engaging with, and understanding children whose parents have a dual diagnosis. Child Adolesc Ment Health.

[48] Kessler RC, Angermeyer M, Anthony JC, Demyttenaere K, Gasquet I, Gluzman S, Gureje O, Haro JM, Kawakami N, Karam A, Levinson D, Medina MME, Oakley BMA, Posada-Villa J, Stein DJ, Adley TCH, Aguilar-Gaxiola S, Alonso J, Lee S, Heeringa S, Pennell B, Berglund P, Gruber MJ, Petukhova M, Chatterji S, Ustün TB (2007). Lifetime prevalence and age-of-onset distributions of mental disorders in the World Health Organization's World Mental Health Survey Initiative. World Psychiatry.

